# Distributional justice and climate risk assessment: An analysis of disparities within direct and indirect risk

**DOI:** 10.1111/risa.17664

**Published:** 2024-10-21

**Authors:** M. J. Anderson, L. Conrow, M. Hobbs, R. Paulik, P. Blackett, T. Logan

**Affiliations:** ^1^ Civil and Natural Resources Engineering University of Canterbury Christchurch Canterbury New Zealand; ^2^ The Cluster for Community and Urban Resilience University of Canterbury Christchurch Canterbury New Zealand; ^3^ Faculty of Health University of Canterbury Christchurch Canterbury New Zealand; ^4^ GeoHealth Laboratory University of Canterbury Christchurch Canterbury New Zealand; ^5^ Earth and Environment University of Canterbury Christchurch Canterbury New Zealand; ^6^ National Institute of Water and Atmospheric Research (NIWA) Wellington New Zealand; ^7^ Urban Intelligence Limited Christchurch Canterbury New Zealand

**Keywords:** climate adaptation, climate risk, distributional justice, indirect risk, natural hazard

## Abstract

Climate change and natural hazard risk assessments often overlook indirect impacts, leading to a limited understanding of the full extent of risk and the disparities in its distribution across populations. This study investigates distributional justice in natural hazard impacts, exploring its critical implications for environmental justice, equity, and resilience in adaptation planning. We employ high‐resolution spatial risk assessment and origin–destination routing to analyze coastal flooding and sea‐level rise scenarios in Aotearoa New Zealand. This approach allows the assessment of both direct impacts (property exposure) and indirect impacts (physical isolation from key amenities) on residents. Indirect impacts, such as isolation and reduced access to resources, have significant adverse effects on well‐being, social cohesion, and community resilience. Including indirect impacts in risk assessments dramatically increases the overall population burden, while revealing complex effects on existing inequalities. Our analysis reveals that including indirect impacts increases the overall population burden, but the effect on inequalities varies. These inequalities can be exacerbated or attenuated depending on scale and location, underscoring the need for decision‐makers to identify these nuanced distributions and apply context‐specific frameworks when determining equitable outcomes. Our findings uncover a substantial number of previously invisible at‐risk residents—from 61,000 to 217,000 nationally in a present‐day event—and expose a shift in impact distribution toward underserved communities. As indirect risks exacerbate disparities and impede climate adaptation efforts, adopting an inclusive approach that accounts for both direct and indirect risks and their [un]equal distribution is imperative for effective and equitable decision‐making.

## 1 INTRODUCTION

To ensure an effective and just approach to climate change adaptation, it is imperative that planners and decision‐makers have the capability to evaluate and comprehend the distribution of risks across communities and broader society (Chu & Cannon, 2021; de Goër de Herve et al., 2023; Lin et al., 2021; Schinko et al., 2023). Assessing the distribution of burdens entails understanding how existing and possible future impacts are distributed, equally or unequally, across communities (Cutter et al., 2008). However, this must be measured, monitored, and communicated across the full suite of burdens resulting from natural hazard events (Best et al., 2023; Logan et al., 2023; Pelling & Garschagen, 2019). Without a comprehensive understanding of how direct and indirect burdens, in this case, property damage and loss of access to essential services, respectively, are distributed within communities, reducing existing inequities and mitigating the anticipated consequences of climate change will be severely constrained and likely to lead to maladaptation (Markkanen & Anger‐Kraavi, 2019).

Indirect risk encompasses the secondary effects that often arise from a primary hazard event, such as disruptions in critical infrastructure, services, and socioeconomic systems (Arrighi et al., 2021; IPCC, 2022; Nicholls & Kebede, 2012). These effects can have far‐reaching consequences, disproportionately affecting populations, and exacerbating existing social, ethnic, and environmental inequalities such as the uneven distribution of risk (Leichenko & Silva, 2014; Markkanen & Anger‐Kraavi, 2019; Schmeltz, 2021). In this paper, we refer to indirect risk as residents that become isolated or “cutoff” from key amenities or locations required to live and thrive as a result of direct damage to the transportation network and amenities themselves. International literature suggests that areas where damage and loss of access have already been experienced are often occupied by those of lesser economic means because they cannot afford to live anywhere else (Beck, 1992; Ratnadiwakara & Venugopal, 2020). Therefore, the ability to identify and evaluate past, present, and future inequalities within direct and indirect risk is essential for informing equitable future decisions and policies such that disproportionately impacted communities receive the necessary support and resources to navigate climate challenges in an equitable and effective manner (Hughes, 2020; Lioubimtseva, 2022; Lioubimtseva & da Cunha, 2023; Shi et al., 2016).

Understanding how inequalities perpetuate due to direct and indirect risk is a key step toward distributional justice, which lies at the core of environmental and climate justice (Low, 2013; McDermott et al., 2013). Climate justice considers local impacts, vulnerabilities, the importance of community voice, and demands for community sovereignty and functioning in both the short‐ and long terms (Schlosberg & Collins, 2014). Similarly, and more widely recognized, environmental justice encompasses three fundamental principles: distributive justice, procedural justice, and recognition, each playing a vital role in achieving equitable (fair) outcomes (Pörtner & Roberts, 2022). Distributional justice addresses the fair allocation of burdens and benefits among individuals, nations, and generations (Doorn, 2015; March et al., 2020; Mohai et al., 2009; Newell et al., 2021). Procedural justice focuses on inclusive decision‐making processes and meaningful participation (Forsyth, 2014; Holland, 2017; Paavola & Neil Adger, 2002; Tomlinson, 2015). Recognition emphasizes the importance of engaging and respecting diverse cultures and perspectives, and acknowledging lived experiences and historic contexts (Fünfgeld & Schmid, 2020). However, despite the critical role environmental and climate justice have in promoting equity, few studies have adequately incorporated these considerations when evaluating the impacts of risk and associated interventions on marginalized groups as discussed in Juhola et al. (2022). This lack of adequate consideration of distributional justice within risk assessment has been increasingly noted within the literature (Jafino et al., 2021; Torres et al., 2020). To bridge this gap, it is essential to develop methodologies that enable a rigorous assessment of the temporal and sociospatial distribution of risks, which again, is crucial for equitable adaptation planning (Shi et al., 2016).

At the intersection of the accessibility and distributional justice literature is that of the transport equity and justice field, drawing connections between transport disadvantage, social exclusion, and accessibility (Di Ciommo & Shiftan, 2017; Lucas, 2012). This growing body of literature emphasizes the need to move beyond traditional cost–benefit analysis approaches and incorporate a broader understanding of justice and equity in transport planning and evaluation (Pereira et al., 2017), aligning with our paper's focus on developing a wider knowledge of distributional justice and community resilience. Recent research has introduced new methodologies, such as the Transportation Justice Threshold Index Framework (Oswald Beiler & Mohammed, 2016), which aim to standardize the identification of transportation justice areas across different jurisdictions. These approaches complement our proposed method by providing additional tools for quantifying and evaluating transport equity. Furthermore, scholars have argued for considering accessibility as a key measure of transport equity, suggesting it should be distributed independently of other goods (Martens, 2012). This perspective supports our emphasis on accessibility as a crucial factor in assessing the fairness of urban systems.

Achieving a more equitable distribution of risk in urban systems requires that adaptation considers communities' various needs and capabilities across time (Gössling, 2016; Loschner et al., 2019; Lucy et al., 1977; Talen & Anselin, 1998). Therefore, for risk‐reduction measures to be successful, we require the ability to assess and communicate the distribution of impacts across different sociodemographic groups such as income, ethnicity, deprivation status, and age among many others. For example, many individuals and collectives around the world with lower incomes have no choice but to live in informal settlements, public housing, or hazardous and high‐risk locations; suffer from preexisting health conditions (Watts et al., 2015); and have fewer resources to prepare for, cope with and recover from stresses and shocks (Mearns & Norton, 2009). These community characteristics are important for several reasons. Traditional views on vulnerability often focus on “susceptibility,” but understanding these characteristics can lead to insights on both higher and lower susceptibility to certain hazards. Moreover, these characteristics can also influence how a community may perceive and react to climate risks, affecting the level of social cohesion and trust in government institutions (Adger et al., 2013). Failure to identify groups living in circumstances that put them at higher risk of negative impacts and support them equitably will likely exacerbate current inequities.

To ensure that current inequities are not exacerbated, decision‐makers must be equipped with the appropriate tools and techniques to identify unequal distributions of burdens across sociodemographic groups (disparities) in future climate scenarios such that decision‐makers can apply their own normative frameworks and local contexts when determining just and equitable outcomes. One technique that can be used is that of community capacity which ensures that the risk to the capacity of each resident, household, or community is not significant. This “community capacity” approach to resilience and adaptation typically requires identifying the characteristics of a community that enable it to withstand, prepare for, recover from, and transform after a shock (Cutter, 2016; Cutter et al., 2010, 2014; Sherrieb et al., 2010). Well‐known examples of this approach to resilience are the resilience indicators such as Disaster Resilience of Place (DROP, Cutter et al., 2008), Baseline Resilience Indicators for Communities (BRIC, Cutter et al., 2014), and Social Vulnerability Index (SOVI, Cutter et al., 2003a). However, to aid current risk assessment approaches that focus solely on direct impacts such as infrastructure failures, we must build upon current community capacity measures to consider the indirect impacts on the people, places, and things that are required to foster the capacities necessary for resilience (Arrighi et al., 2021; Khazai et al., 2013; Lioubimtseva & da Cunha, 2023; Logan et al., 2022). By considering these impacts, we enable the understanding of how infrastructure can enable these capacities within communities before, during, and after a disruption.

Existing studies demonstrate the ability of infrastructure to enable resilience‐enhancing capacities by detailing how community resilience depends on equitable access to essential services and amenities (Logan & Guikema, 2020). Healthcare, education, food, emergency services, and cultural and recreational centers are examples of amenities that are essential to the livability, safety, and cohesion of a community (Contreras et al., 2017; Dempsey et al., 2011; Talen, 2003; United Nations Educational, Scientific and Cultural Organization & World Bank, 2018; Winter & Farthing, 1997). Persistent disruption of access to these amenities—a type of secondary stressor or indirect impact—can transform a short‐term event into a long‐term social disaster (Contreras et al., 2017; Lock et al., 2012; Netter, 2016; Watt, 2017). For example, flooding can cause significant social and mental health problems that can continue over extended periods of time, specifically, those who experience a higher disruption to daily routines have been shown to present with higher levels of mental health problems (Fernandez et al., 2015). However, in the presence of sufficient and equitable access, communities begin to foster a range of positive capacities such as community cohesion, social capital, and sense of place (Forrest & Kearns, 2001; Jennings & Bamkole, 2019). All of these qualities can reduce the severity of other long‐term stresses and, in doing so, enhance a community's ability to withstand, prepare, and act following a disruption, thus improving the community's resilience.

Indirect risks within the context of natural hazards and climate change have emerged as critical considerations in understanding the full scope of vulnerabilities and impacts within communities (Hochrainer‐Stigler & Reiter, 2021; Reiter et al., 2022). While studies that quantify the indirect impacts of infrastructure failure on lives, livelihoods, and economies are limited as noted in IPCC (2022), some indirect impacts, such as access disruption, are becoming more common within infrastructure and community risk assessments (Anderson et al., 2022; Jasour et al., 2022; Jafino et al., 2020; Logan et al., 2023; Lan et al., 2023). However, the distribution of burdens among both direct and indirect risks is rarely considered. Given recent studies have found that including indirect risk significantly increases the population impacted, we must evaluate the consequential shift in the distribution of these impacts. Logan et al. (2023) and Jasour et al. (2022) also found that an increase in the number of people impacted was paired with the decrease in lead time for when these impacts may occur. In some cases, communities that are planning for the direct impact of sea‐level rise may be indirectly impacted decades sooner than they anticipate (Logan et al., 2023). Therefore, it is imperative that decision‐makers understand these increases and temporal shifts in burdens as well as the distribution among different population types so that adaptation can be timely, equitable, and effective (Pelling & Garschagen, 2019).

Our objective is to enhance the understanding of the direct and indirect impacts of natural and climate‐influenced hazards, as well as explore their contribution to existing and future disparities. This is motivated by the notably limited research on the indirect effects of climate change (IPCC, 2022) and the growing concern over how climate change is worsening inequalities (Markkanen & Anger‐Kraavi, 2019). The objectives of this paper are to (1) develop and assess a methodology to evaluate the distribution of burdens within a spatiotemporal variant risk assessment and (2) evaluate how the distribution of risk changes when considering both direct and indirect impacts. Specifically, we seek to understand whether existing disparities seen within the distribution of direct impacts are exacerbated when indirect impacts are also considered. To do this, we consider coastal inundation (including changes with rising sea levels and annual recurrence intervals) and evaluate the direct and indirect impacts on different sociodemographic groups. This enables us to evaluate how impacts are distributed between ethnic, economic, and social groups both now and across future climate scenarios and therefore enable decision‐makers to consider these results in conjunction with their own normative frameworks and local contexts when determining just and equitable outcomes.

To demonstrate this methodology and explore the distributional justice of risk across direct and indirect impacts, we use New Zealand as a case study. Aotearoa New Zealand is particularly exposed to coastal hazards due to its extensive coastline and concentrated coastal populations. The country's diverse sociodemographic landscape provides a rich context for examining how climate risks intersect with existing social and economic inequalities. New Zealand's population comprises various ethnic groups, including New Zealand European (Pākehā), Māori (the indigenous people), Pacific, Asian, and other communities, with varying socioeconomic statuses across urban and rural areas. The indigenous Māori context is especially significant, given the historical and ongoing impacts of colonization, which have led to socioeconomic disparities that may exacerbate vulnerability to climate‐related risks. New Zealand's multilevel governance structure, with its division between territorial authorities and regional councils, adds complexity to adaptation planning and implementation. By applying our methodology to this varied sociocultural and governance landscape, we aim to provide insights that can inform the country's adaptation strategies, particularly in light of recent national‐level initiatives such as the National Climate Change Risk Assessment and subsequent Adaptation Plan, while contributing to the global understanding of distributional justice in climate risk.

## 2 METHODS

Understanding the changing dynamic of disparities between direct and indirect risk requires determining the number of exposed and isolated residents due to different hazard events under different return intervals and scenarios. This requires evaluating the access of residents to essential services in the event of each hazard scenario. In this study, we evaluate the network distance between a set of origin–destination (O–D) pairs after a series of simulated coastal flood events where transport links and amenity locations are removed based on their exposure and vulnerability to the hazard (Anderson et al., 2022; Logan et al., 2019, 2021).

Using this information, we assess the burden for both direct (property exposure) and indirect (service isolation) impacts at various scales throughout New Zealand for a number of different sociodemographic groups. The information has been sourced at the statistical area 1 (SA1) level. SA1 units are the smallest census spatial unit used within New Zealand with a typical population between 100 and 200. The sociodemographic groups considered include:

1. 2018 New Zealand Deprivation Index (Deciles 1–3, 4–7, and 8–10). The New Zealand Deprivation Index is an area‐based measure of socioeconomic deprivation. It is based on nine Census variables such as employment, education, and living situation. Areas in Decile 1 represent places with approximately 10% of the population that is the least socioeconomically deprived, while Decile 10 represents the most socioeconomically deprived areas of New Zealand (Atkinson et al., 2019).
2. Population grouped by household income ($0–40,000, $40,000–90,000, $90,000+). Household income is sourced from the 2018 New Zealand census at the SA1 level (Stats NZ, 2018).
3. Ethnicity (European, Māori, Pacific, Asian, MELAA [Middle Eastern, Latin American and African ethnicities], and Other). Ethnicity is sourced at the SA1 level as the total population of each ethnic group from the 2018 New Zealand census (Stats NZ, 2018).

### 2.1 Data inputs and processing

To complete this analysis, we use the following data for the entirety of New Zealand at the finest spatial resolution possible:

1. Transportation network (OpenStreetMap contributors, 2017). A spatial and graphical representation of New Zealand's road transportation network as of 2023.
2. 2018 Spatial demographic data: The usually resident population, NZ Index of Socioeconomic Deprivation, ethnicity, and household income at the SA1 level (Stats NZ, 2018; Atkinson et al., 2019).
3. Building outlines and residential property rating valuation data (CoreLogic, 2020; Land Information New Zealand, 2021b).
4. Amenity and essential service locations (supermarkets, pharmacies, primary schools, hospitals and medical centers, and emergency services) (Wiki et al., 2020; Land Information New Zealand, 2021a; Ministry of Education, 2021; OpenStreetMap contributors, 2017).
5. Coastal flooding data for 189 different scenarios including various exceedance probabilities and sea‐level rise scenarios (10 and 30 m resolution scenarios for annual exceedance probabilities of 50%, 20%, 10%, 5%, 2%, 1%, 0.5%, 0.2%, and 0.1%, each for sea‐level rise increments of 10 cm between 0 and 2 m) (Paulik et al., 2023).

Current approaches that measure access and property exposure at the SA1 level exhibit limitations in capturing the nuanced distribution of risk and accessibility because the information is representative of the statistical reporting area instead of the household level. To explore this at a household level, we use dasymetric mapping techniques. By disaggregating data from coarse geographic units to the household level, dasymetric mapping enables a more nuanced assessment of how different segments of the population are exposed to risks associated with natural hazards and their proximity to essential services and infrastructure.

Therefore, to improve the accuracy of our study, we employed a dasymetric mapping technique that disaggregates the study population from the SA1 level to individual buildings by considering the relative footprint area of each building (Mennis, 2009). To attain household‐level populations, we calculated the relative footprint area for each building identified as residential use from CoreLogic (2020) by dividing its footprint area by the total footprint area of all buildings within each SA1 unit. This calculation provided a proportion indicating the spatial significance of each building based on its footprint area. Next, we allocated the population of each SA1 unit to the corresponding buildings using the relative footprint area. The population of each SA1 unit was multiplied by the relative footprint area of the buildings within that unit to determine the population allocation. The equation used for this is

(1) $${\hat{Y}}_{t}=Y_{s}*\left(\frac{A_{t}{\hat{D}}_{c}}{\sum_{t\in s}A_{t}{\hat{D}}_{c}}\right),$$

where ${\hat{Y}}_{t}$ is estimated population of building t; $Y_{s}$ is observed population of SA1 unit s; $A_{t}$ is area of building footprint t; and ${\hat{D}}_{c}$ is estimated population density of class c.

While dasymetric mapping is a valuable technique for refining the spatial representation of data, it does have limitations that should be acknowledged. These limitations arise due to assuming an equal distribution of population across the total building footprint in the SA1. While there may not be significant variation within the SA1 (with 100–200 people), this still introduces error in the estimates of the sociodemographic characteristics of a household. For instance, household overcrowding in individual units is not considered and can be of increased prevalence in certain cultural or socioeconomic groups (Baker et al., 2012). In addition, by assuming an equal distribution of population types within each home, dasymetric mapping may oversimplify the complex dynamics of household composition, potentially leading to the misrepresentation of disparities. Moreover, dasymetric mapping relies on static land‐use classifications, which can result in nonresidential buildings being associated as homes. This misclassification can distort the understanding of population distribution and risk patterns, particularly in mixed‐use or multifunctional areas. We limit this by pairing both the Land Information New Zealand (2021a) building outlines with the identified CoreLogic (2020) residential land‐use parcels.

**FIGURE A.2:**
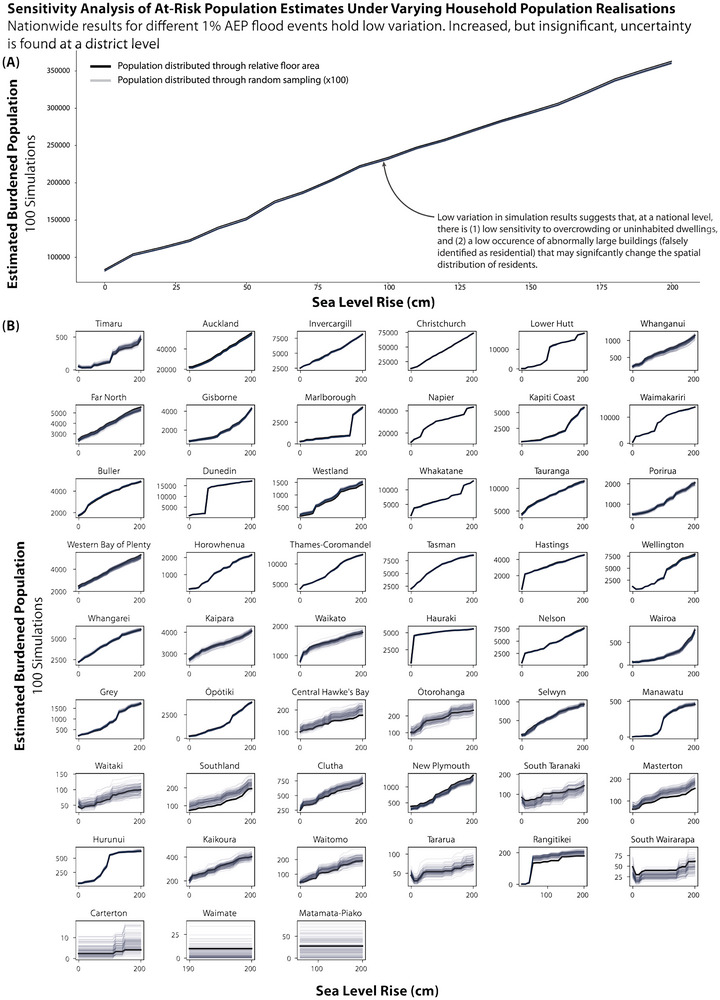
Sensitivity analysis of at‐risk populations based on varying population distributions from the statistical area 1 (SA1) level to the building level at a (A) national scale, and (B) district scale.

To evaluate the impact of this error on the results, a sensitivity analysis was conducted to assess the robustness of our dasymetric mapping approach. The objective was to understand how our results may differ when compared to alternative realizations for the population distribution sampling at an SA1 level. We performed a random sampling procedure, generating 100 samples of building‐level populations from the SA1‐level population data. Then, to compare the results, we analyzed the number of impacted residents with the relative footprint area method versus population distributions obtained from a random sample methodology. This allowed for the identification of any significant variations or patterns that emerged from the sensitivity analysis. Where large variations at the SA1 level were found, a visual inspection was completed to confirm the appropriate classification of building use. The final results of the sensitivity analysis are plotted at a national and district level and are shown in Appendix A.2. Figure A.2 illustrates the comparison between the population distribution derived from the dasymetric mapping approach and the population distributions resulting from the 100 random samples. This comparison shows that the chosen methodology is largely consistent with any variation seen across the 100 random samples at both a nationwide and a more localized scale, suitably justifying the use of dasymetric mapping despite its common trade‐offs.

**FIGURE 1:**
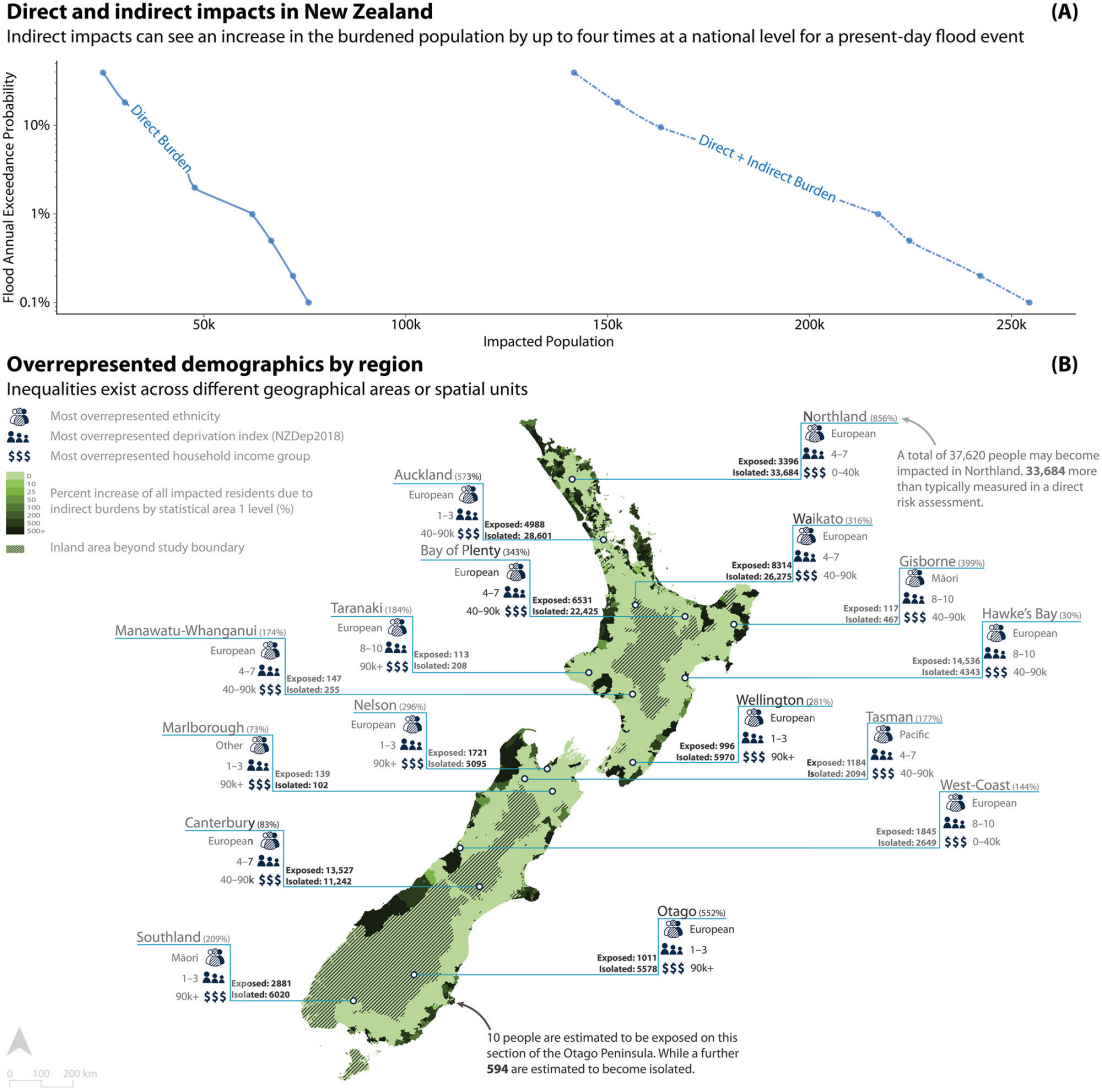
Panel A demonstrates the population impacted by direct and indirect impacts after a coastal flood of varying return intervals with 0 cm sea‐level rise. Panel B shows, (1) Chloropleth map: Variation in the inclusion of indirect impacts at a statistical area 1 scale by the full study population, and (2) Regional statistics: The subsociodemographic groups most disproportionately impacted at the regional and unitary council level. These results show a one in 100‐year flooding scenario with 0 cm sea‐level rise. Socioeconomic deprivation index of 8–10 indicates a high level of deprivation, while 1–3 indicates a low level of deprivation. Note that Appendix A.1 demonstrates the local spatial diversity that can be seen between direct and indirect impacts at a household level.

**FIGURE 2:**
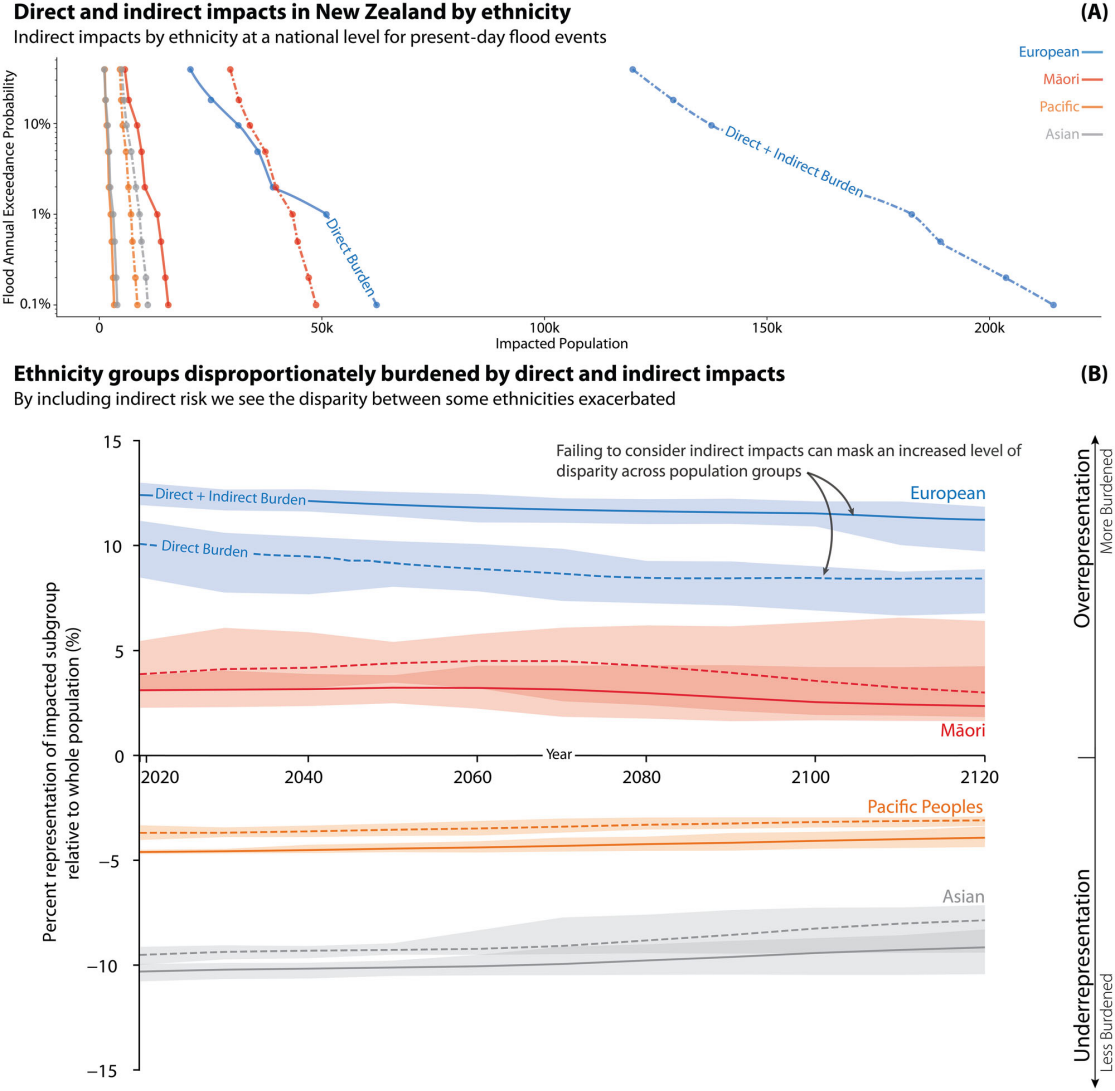
Panel A demonstrates the population impacted by direct and indirect impacts after a coastal flood of varying return intervals with 0 cm sea‐level rise. Panel B shows the under‐ and overrepresentation of New Zealand's largest ethnicities impacted by direct and indirect burdens over time. The uncertainty bounds in Panel B represent the variation in results driven by expected sea levels under SSP1 to SSP5.

**FIGURE 3:**
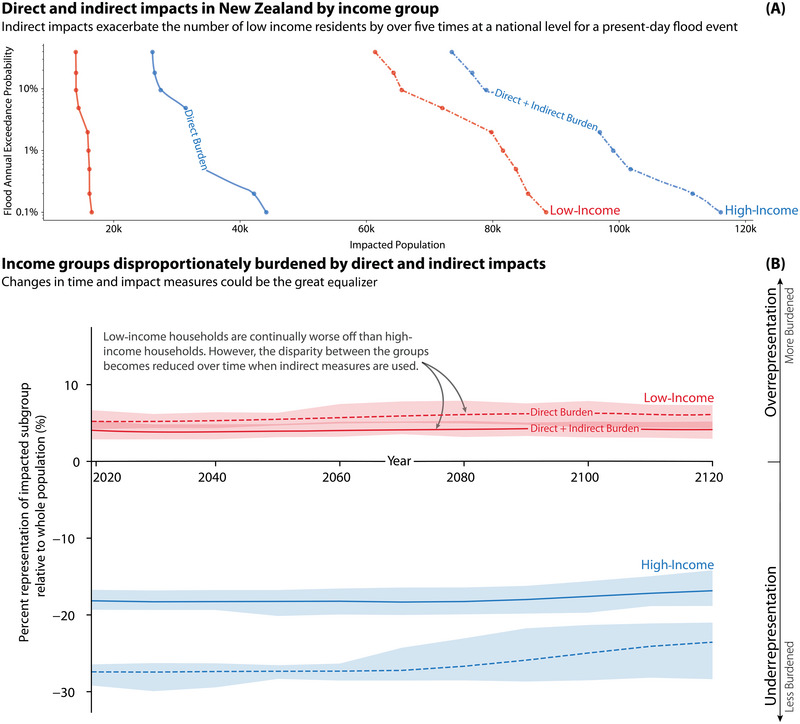
Panel A demonstrates the population impacted by direct and indirect impacts after a coastal flood of varying return intervals with 0 cm sea‐level rise. Panel B shows the under‐ and overrepresentation of high ($90,000+) and low ($0–40,000) income populations impacted by direct and indirect burdens over time. The uncertainty bounds in Panel B represent the variation in results driven by expected sea levels under SSP1 to SSP5.

**FIGURE 4:**
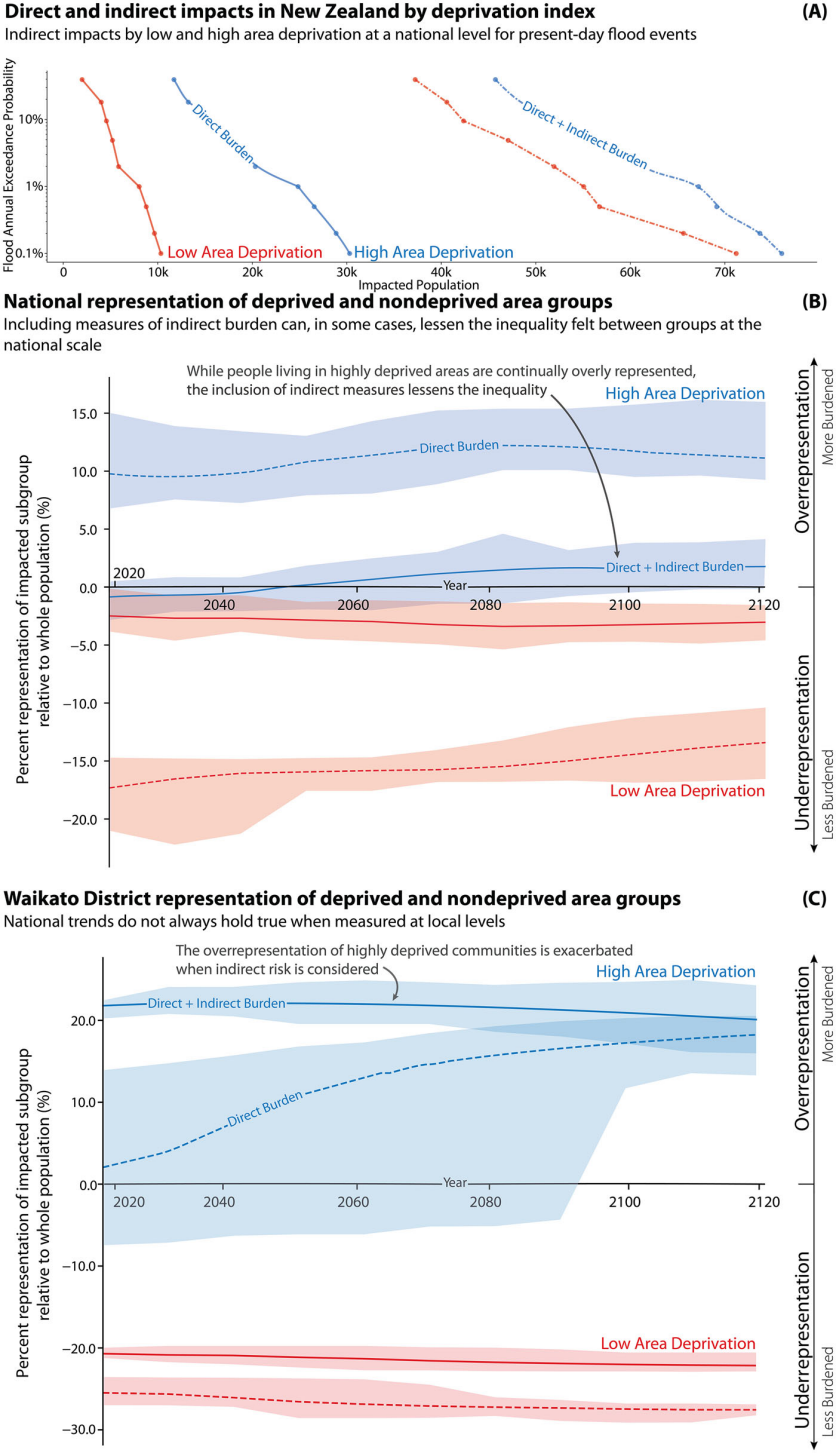
Panel A demonstrates the population impacted by direct and indirect impacts after a coastal flood of varying return intervals with 0 cm sea‐level rise. Panel B shows the under‐ and overrepresentation of high (8–10) and low (1–3) socially deprived populations impacted by direct and indirect burdens over time and at a national level. Panel C shows the under‐ and overrepresentation of high (8–10) and low (1–3) socially deprived populations impacted by direct and indirect burdens over time and at a district level. The uncertainty bounds in Panels B and C represent the variation in results driven by expected sea levels under SSP1 to SSP5.

### 2.2 Evaluating direct impacts

Direct impacts are considered as the physical exposure that a hazard has on various people and infrastructures. This includes, but is not limited to, roads, amenities, and residential homes. The direct impact is calculated by spatially overlaying each asset with the flood extent and reporting the maximum estimated flood depth within the provided area. Predetermined flood depth categories of inundation relating to Table 1 were used to polygonize each hazard scenario within Google Earth Engine, as rasterized hazard data were prohibitively computationally intense at a national scale. In addition, the spatial extent of the high‐resolution (10 × 10 m) hazard data did not cover areas where LiDAR data have not been collected. These areas were supplemented with a lower resolution (30 × 30 m) data set produced from bias‐corrected Shuttle Radar Topography Mission (SRTM) data.

**TABLE 1:** Operability thresholds.

Asset type	Inoperable depth	Reference
**Road**	300 mm	Pregnolato et al. (2017)
**Property**	Any exposure	—
**Hospital**	400 mm	Santhanam (2021)
**Medical clinic**	Any exposure	—
**Supermarket**	Any exposure	—
**Primary school**	Any exposure	D'Amato and Preston (2020)
**Fire station**	300 mm	Pregnolato et al. (2017)

Once polygonized, each road, building, and amenity was intersected with the hazard to determine the exposure value. This value then determines whether the asset is likely to be operable during and immediately after the flood event. Flood depth values used to determine the operability status can be seen in Table 1.

The exposure analysis in this study is subject to certain limitations. These limitations primarily stem from the nationwide collation of digital elevation maps, which impacts the accuracy of hydrologic and hydraulic features representation, including flood mitigation actions, stormwater infrastructure, and stopbanks. Future studies could incorporate more detailed local and regional data to address these limitations and provide more precise results. Further information on the methods used to create the coastal flooding scenarios can be found in Paulik et al. (2023).

### 2.3 Evaluating indirect impacts

Evaluating indirect impacts is determined by factors beyond hazard exposure that make residential properties [un]inhabitable. In addition to not being directly exposed to a hazard event, homes must also have adequate access to various services (e.g., food, employment, healthcare, etc.). Therefore, to include indirect impacts, we must understand how access from the home to key services may change after a flooding event. Similar to Logan et al. (2023), we consider a household isolated if it cannot reach at least one amenity category across supermarkets, pharmacies, primary schools, hospitals and medical centers, and fire stations via the road network. That is, if a household cannot access a healthcare provider, supermarket, pharmacy, primary school, or fire station, they are considered indirectly impacted. While other forms of transport are available such as marine and air, in the context of this study only road‐based transportation is considered. In addition, this measure of isolation assumes that all amenities have the same impact and that the consequences of isolation are the same across all regions and population groups. This equal treatment of isolation may mask inequities if used across multiple contexts without the acknowledgement of the varying needs of different households, as the relative importance of losing access to specific amenities can vary depending on the local context and the needs of different population groups.

#### 2.3.1 O–D pairs

To evaluate access, we must first establish the O–D pairs. Origins, the point from which access is evaluated, are defined in this study by all residential building centroids with an estimated population greater than 0. To reduce the computational demand of the access analysis, we only take origins that are within a 50 km buffer of the largest coastal flooding scenario extent.

Destinations are the centroids of amenities and services that most residents would either rely upon daily or weekly, or that provide emergency relief as and when needed. This study includes supermarkets, pharmacies, primary schools, hospitals and medical centers, and fire stations. Although fire stations are here referred to as a destination, this provides an estimate for the ability of any given household (origin) to be reached by fire emergency services.

Again, to reduce computational demand, we create and filter a list of O–D pairs. To filter this array for each amenity type, we measure the distance to either (a) all amenities within 10 km or (b) the closest 15 amenities (by euclidean distance). Then, we report the shortest network distance from the origin to each amenity type.

#### 2.3.2 Measuring access

Access has numerous dimensions (see Penchansky & Thomas, 1981; Saurman, 2016); however, in this case, we measure and discuss “access” as proximity via the road network to operational services. Using the aforementioned filtered array of O–D pairs, we use Open Source Routing Machine (OSRM) to query the driving network distance between points (Luxen & Vetter, 2011) which, as per Logan et al. (2019), removes the use of footpaths, footbridges, and similar pedestrian access routes. The returned array of distances can then be used to find the distance to the nearest destination (of each type) for each origin. Where OSRM returns a nil distance between an origin and a destination type, the origin is isolated from that service (Anderson et al., 2022). The OSRM model is calibrated by completing all O–D queries on a non‐hazard‐impacted network to ensure all O–D pairs are viable, this eliminates data quality or routing issues as causes of future nil distance results.

The routing analysis conducted in this study is based on the current road network and does not account for potential future adaptive measures that could enhance existing routes or introduce alternative routes. In addition, it is subject to any limitations or inaccuracies from the transportation network graph methodologies within OpenStreetMap contributors (2017). It is important to recognize that this analysis serves as a starting point to identify areas that may require intervention but should be complemented by considering future adaptation and growth pathways. In addition, the population levels used in this study rely on current census data and do not project into the future. As coastal areas may experience both retreat and growth, future work should incorporate projections to capture the dynamic changes in population distribution.

#### 2.3.3 Operational status of destinations and road segments

To understand the risk of isolation to varying services under different coastal flooding scenarios, we must repeat the above steps to query residents' access on a modified transportation and service network. As per Anderson et al. (2022), we remodel the transportation network in OSRM by removing all links that experience 300 mm of inundation or greater. Three hundred millimeters is the average depth at which a passenger vehicle starts to become buoyant and is therefore widely recognized as the safety threshold for most passenger vehicles (Pregnolato et al., 2017). In addition, to determine the operability of services, we used varying depths that correspond to the likelihood of operation. These can be seen in Table 1. Where an exposure intensity threshold (inoperable depth) is unknown we assume operability will fail on any exposure of the hazard.

Bridges are excluded from the road network when assessing flooding impacts due to limitations in available data such as bridge deck heights (so bridges are assumed to remain passable). This assumption is unlikely to significantly affect the overall results given that inundation to the ramps/connecting roads is still considered.

### 2.4 Evaluating disparities

To demonstrate how risk can become disproportionately distributed among various groups and exacerbated when considering indirect risk, we show the relative representation for different sociodemographic groups (ethnicity, income, and socioeconomic deprivation) although acknowledge there are other measure of spatial equity available (Whitehead et al., 2019). By evaluating a group's risk over time, we can better identify when (and where) it is appropriate to engage the group in adaptation discussions. More importantly, the engagement can be tailored to the specific needs of the identified group.

To evaluate disparities in different sociodemographic groups and understand the over‐ or underrepresentation in the impacted population, we implemented the following methodology: First, we calculated the subset sizes (*n*) within each sociodemographic group for both the burdened and business‐as‐usual (nonimpacted prehazard) populations. Using Equation (2), we then computed the percent representation (r) for each sociodemographic group across different areas and spatial scales. This equation is resolution agnostic and can be used at any spatial scale. The percent representation value (r) shows the relative representation of each sociodemographic group within the impacted (burdened) population compared to the business‐as‐usual population.

Interpreting the results involved analyzing the calculated percent representations for each sociodemographic group. A positive r value indicated an overrepresentation of the sociodemographic group within the impacted population compared to the business‐as‐usual population. Conversely, a negative r value signified an underrepresentation of the sociodemographic group in the impacted population. The magnitude of r reflects the extent of the disparity.

(2) $$r=100*\left(\frac{n_{burdened}}{N_{burdened}}-\frac{n_{bau}}{N_{bau}}\right),$$

where r is percent representation relative to original sample, N is sample size, n is subset of sample size, bau is business‐as‐usual population, and burdened is impacted population.

It is important to note that ethnicity data in the New Zealand Census allow for multiple ethnic identifications, meaning that an individual can identify with more than one ethnic group. As a result, the total population across all ethnic groups may add up to more than 100% of the total population. To account for this, when calculating the percent representation (r) for each ethnic group, the subset sizes (n) and sample sizes (N) are based on the total responses within each ethnic group, rather than the total number of individuals. This approach ensures that the relative representation of each ethnic group is accurately captured, even when individuals identify with multiple ethnicities.

Equally, limitations exist when using household income groupings, as household income does not account for differences in household size and composition, which can affect the distribution of economic resources among household members. Second, total household income does not capture income disparities within households, such as those related to gender or age. Third, the cost of living varies across different regions, and household income does not account for these differences, which can affect the purchasing power and living standards of households. Despite these limitations, grouping the population by household income still provides insights into the potential disparities in coastal flooding risk across different socioeconomic groups. However, the results should be interpreted with these limitations in mind, and future studies could benefit from using equivalized income or other more comprehensive measures of socioeconomic status, if available.

Evaluating disparities over time requires an understanding of the localized relative sea‐level rise at each location around the country. This can be achieved by combining the effects of vertical land movement (VLM) and the rate of local mean sea‐level rise. VLM, which encompasses processes such as subsidence or uplift, can significantly change the rate of relative sea‐level rise and see communities impacted far sooner than anticipated when compared to global rates of mean sea‐level rise. By integrating Naish et al. (2022) VLM data with the five IPCC (2022) shared socioeconomic pathways (SSPs), an estimate of localized relative sea‐level rise can be achieved over time. VLM has been mapped around New Zealand's coastline at approximately 2 km spacing. Using this information, we associate each household with an approximate rate of VLM. Each household can then be given an estimated relative sea‐level rise associated with a year and SSP. This allows the evaluation of risk and its disparity over time, additionally, the use of SSPs allow for temporal uncertainty to be measured based on the variation expected in sea‐level rise over time. However, it should be noted again that population and infrastructure are assumed constant over this period due to the uncertainty associated with land use and population distributions over long time periods. That is, this analysis intends to provide decision‐makers impetus on the cost of “doing nothing.”

## 3 RESULTS

The results presented in this section demonstrate how the introduction of indirect risk drives a significant increase in the impacted population and an unequal exacerbation of risk across different sociodemographic groups. Figure 1A (graph) shows a significant increase from 61,993 to 217,002 in the number of people at risk nationally when considering isolation as an indirect impact for a present‐day coastal flood scenario. Figure 1B highlights (1) the spatial differences that arise from taking indirect impacts into account across the country on an SA1 level, and (2) which sociodemographic groups are most overrepresented within the impacted population in each region. The groups most overrepresented vary based on geographic location. This variation emphasizes the spatial dimension of distributional justice and the need for tailored interventions between regions.

Table 2 shows the impacted populations by focusing on the nationwide impacts as well as the top five and bottom five territorial authorities (local municipalities). These territorial authorities are ranked based on the percentage increase in the impacted population, considering both direct and indirect impacts. The table provides an overview of how different areas are affected, demonstrating that some districts may see an increase of over 2000% in the total burdened population when considering both direct and indirect burdens. Finally, Table 2 identifies which sociodemographic groups are most overrepresented as per Equation (2). Table 2 also shows the number of people burdened by each category. A full set of results can be found in Table A.1.

**TABLE 2:** Summary of results by territorial authority. Results are shown for a one in 100‐year coastal flooding event with 0 cm sea‐level rise.

Area	Study population	Exposed population	Isolated population	Total impacted population	Total percent increase	Most overrepresented		
						Ethnicity	Deprivation index	Household income
**Coastal New Zealand**	**4,414,410**	**61,993**	**155,009**	**217,002**	**250**	**European**	**4–7**	**40–90k**
**Waitaki District**	22,268	1	91	92	9100	European	4–7	40–90k
**Wairoa District**	8849	6	412	418	6866.7	Māori	8–10	40–90k
**South Wairarapa District**	11,381	3	81	84	2700	European	4–7	40–90k
**Otorohanga District**	5724	16	367	383	2293.8	Māori	8–10	0–40k
**Whangarei District**	94,599	715	14,544	15,259	2034.1	European	1–3	40–90k
**…**								
**Hastings District**	79,520	1710	727	2437	42.5	European	4–7	40–90k
**Waimakariri District**	61,157	2136	804	2940	37.6	Māori	8–10	40–90k
**Hauraki District**	15,917	3491	945	4436	27.1	European	4–7	40–90k
**Napier City**	64,016	12,761	3177	15,938	24.9	Māori	8–10	40–90k
**Horowhenua District**	31,304	105	11	116	10.5	European	4–7	40–90k

### 3.1 Examining the distribution of burdens

Building upon the summary provided by the previous section, we now examine how the representation of each sociodemographic group changes when considering direct and indirect impacts. The aim is to further investigate the potential effects of including indirect impacts on ethnicity, deprivation, and income disparities over time. Using SSP scenarios and accounting for localized VLM, Figures 2, 3, 4 examine how including indirect impacts over time influences current inequalities.

#### 3.1.1 Ethnicity

We begin by presenting the results of our analysis, highlighting the disparities experienced by various ethnic groups. Specifically, we assess the proportion of each sociodemographic group relative to the full population (both as a whole and of the impacted subgroup) for both direct and indirect burdens. Using this information, we can understand whether each group is overrepresented (relatively more burdened) or underrepresented (relatively less burdened) for any given hazard scenario. Importantly, the change in representation can be quantified for each group to understand whether a specific group's representation is exacerbated or reduced by including indirect impact measures.

Figure 2 demonstrates that when compared to the relative distribution of the full population, European and Māori populations are consistently overrepresented within the impacted group, while Pacific and Asian populations are consistently underrepresented. When transitioning from considering only the direct impacts to also including indirect impacts, we observe that the degree of over‐ or underrepresentation becomes more pronounced for all groups but Māori which see an attenuation of the magnitude of representation within the impacted population. In other words, Figure 2B shows that underrepresented sociodemographic groups experience fewer changes in impacts compared to overrepresented populations whose impacts can grow disproportionately. Two other ethnic groups, MELAA and other, are not shown in Figure 2 due to both groups neither being over‐ or underrepresented.

#### 3.1.2 Income

In this section, we focus on the disparities experienced by high‐ and low‐income populations. The low‐income sociodemographic group is initially overrepresented within the impacted group, indicating a higher burden relative to their proportion in the full population. Including indirect impact measures slightly reduces the level of overrepresentation for the low‐income group, bringing them closer to an equal sharing of burdens. However, a large disparity still exists between the two groups.

#### 3.1.3 Area‐level socioeconomic deprivation

Now we focus on the disparities observed among populations living in areas of high and low‐level deprivation indices. To determine what sociodemographic groups living in different area deprivation indices are over‐ or underrepresented in the exposed population, we evaluate the exposed population as a proportion of each full, nonexposed, sociodemographic group. Our findings reveal an opposite trend compared to the ethnicity analysis, where the evaluation of both direct and indirect impacts sees the distribution of burdens across the different deprivation groups attenuated rather than exacerbated at the national level. Specifically, communities in high‐deprivation areas are overrepresented in the directly impacted group but by including indirect impacts brings this sociodemographic group closer to a more equal sharing of burdens with the lower deprivation group.

However, in Figure 4C we note that national trends of attenuation are not always seen at localized scales. In the Waikato District, the inclusion of indirect impacts exacerbates the inequality between communities living in areas of high‐ and low‐socioeconomic deprivation. The distribution of burdens is also seen to change over time in the population living in areas of high socioeconomic areas whereby 2130 the magnitude of overrepresentation grows from 2% to 20%.

The analysis of distributional justice in relation to high and low‐socioeconomic deprivation indices is subject to a moderate level of uncertainty, as depicted by the large bounds observed in our results. This range of results arises from the consideration of different return interval events and SSPs.

## 4 DISCUSSION

The objective of this study was to examine distributional justice in the context of natural hazard impacts and assess how the distribution of burdens vary across time, spatial location, spatial scale, and sociodemographic groups. Specifically, this paper explored a methodology to evaluate [in]equality within a risk assessment and understand the distributional justice when considering both direct and indirect risk. Current approaches that use conservative measures of risk as direct impacts may occasionally consider the distribution of burdens among a community. In this study, whilst only considering the distribution of direct impacts at a national scale, we note that traditionally underserved populations, such as those with high deprivation, low income, and Indigenous communities, are already overrepresented among those directly affected by coastal flooding. However, when indirect impacts are considered, the distribution of inequality is seen to be highly place‐ and scale‐specific, with the unequal distribution of burdens being attenuated for different sociodemographic groups at a national level when including indirect impacts, but potentially exacerbated at a localized level.

While this study presents findings relative to coastal flooding in New Zealand, the method can be applied to different hazard, geographic, and societal contexts. The method is transferable across all spatially explicit hazard types and could be used for both chronic (e.g., groundwater rise or erosion) and acute (e.g., wildfire, landslides, or ground‐shaking) hazards. The methodology can be modified to different societal contexts where different sociodemographics and amenity types may be more suited. For example, age can be an important indicator of vulnerability and would see different interventions taken depending on the average age of an impacted area. In the case of amenities, while we utilize fire emergency services, healthcare, supermarkets, and education amenities, this could equally be completed with polling locations during an election period or drinking water distribution sites for communities without networked potable water. Regardless of the context, this method provides researchers and practitioners with another resource to explore beyond the direct impacts captured by current approaches.

Current approaches that use conservative measures of risk as direct impacts may occasionally consider the distribution of burdens among a community. In this study, while only considering the distribution of direct impacts at a national scale, we note that underserved populations, such as those with high deprivation and low income, as well as Indigenous communities, are already overrepresented among those directly affected by coastal flooding. However, the dynamics become more intricate when indirect impacts are taken into account.

Including indirect impacts significantly increases the burdened population, in some cases by several orders of magnitude. However, it is important to acknowledge that the definition of isolation used in this study, which considers a household isolated if it cannot reach at least one of the five amenity types (supermarkets, pharmacies, primary schools, hospitals and medical centers, and fire stations), has implications for the absolute results presented where the number of amenity types considered will influence the magnitude of indirect impacts. Regardless, this finding emphasizes that a sole focus on direct impacts will underestimate the extent of the population affected. Using just one coastal flooding scenario, Figure 1B demonstrates at the national and regional scale how widespread the underestimation of risk can be when considering just direct impacts. These findings follow similar trends to research about coastal states in the United States of America (Logan et al., 2023). Almost all regions across New Zealand see the number of people impacted at least doubled. For example, in the Northland Region of New Zealand, an area with an age profile that is older than the national average and a lower average household income than the majority of the country, the number of indirectly impacted (or previously unidentified) residents is over three times that of the directly impacted population (Centre for Social Impact, 2018). This shows an increase of 33,684 people who would previously be omitted from adaptation considerations, Appendix A.1 demonstrates these results and the localized spatial variation that can be seen between direct and indirect impacts. These results highlight the importance of considering indirect impacts in risk assessments.

Identifying indirectly impacted communities is necessary because direct and indirect impacts require different engagement and support when compared to only those directly impacted. The burden of isolation at the community or household level can have cascading impacts that span across various domains. From a psychological perspective, prolonged isolation can contribute to feelings of loneliness, depression, and anxiety, as well as a sense of disconnection and reduced self‐esteem (Fernandez et al., 2015; Lock et al., 2012). These psychological impacts can further erode overall well‐being and hinder individuals' ability to cope with stressors. In addition, community or household‐level isolation can lead to reduced social cohesion, weakened community bonds, and limited opportunities for collective action and engagement (Jennings & Bamkole, 2019). This can hinder community resilience, decision‐making processes, and overall community well‐being.

Isolated households also encounter barriers in accessing the necessary resources and services required for continued function and fostering community resilience, reducing their adaptative capacity for future events (Lucas, 2012). For example, lifeline infrastructures such as electricity, potable water, and wastewater are often colocated with the transportation network such that residents that are isolated from key amenities may also be impacted by the loss of utility service (Allen et al., 2019).

The realized burden of direct and indirect impacts is highly dependent on the characteristics of the community affected (Cutter et al., 2003b; Engle, 2011). Therefore, considering indirect risk (as well as direct risk) is important for ensuring that a nation's or community's climate adaptation strategy is just. As introduced earlier, environmental justice includes distributional, procedural, and recognitional justice. Although this paper is primarily focused on distributional justice, there are important implications for both procedural and recognitional justice. For instance, when national entities allocate adaptation funding to regions and communities based on their identified risk profiles (Ministry for the Environment, 2022), an approach that solely considers direct risks may result in an unequal distribution of resources, potentially overlooking areas facing substantial indirect risks. Failing to account for the disparities experienced by different groups not only raises concerns about distributional justice but also recognitional justice, as it may neglect the unique needs and vulnerabilities of certain communities. Furthermore, the decision‐making processes involved in these allocations are a matter of procedural justice, emphasizing the importance of inclusive and transparent procedures that consider the voices and perspectives of all affected parties.

For instance, consider the practice of allocating adaptation funding from the central government level to regions and communities based on their identified risk profile (Ministry for the Environment, 2022). An exclusive focus on direct risk within this approach may lead to a disproportionate allocation of resources to areas with lesser risk. If this fails to consider different groups and the disparities, there is clearly an issue for distributional justice, but also recognitional justice. How these decisions are made pertains to procedural justice.

Another aspect where procedural justice becomes critical is specific intervention options such as managed retreat. Managed retreat is often considered an adaptation option for at‐risk communities because homes may no longer be habitable. This evaluation of habitability is conventionally based solely on direct impacts. This focus overlooks the critical dimension of accessibility to amenities and emergency services. Access to these amenities/services is considered by the United Nations' Universal Rights as a critical factor for housing (United Nations General Assembly, 1949). The exclusion of indirect impacts, like isolation, in risk assessments has the potential to significantly distort discussions regarding managed retreat strategies. This oversight not only raises questions about distributive justice by leaving many unsupported but also challenges the principles of procedural justice by potentially excluding thousands of affected residents from decision‐making processes and disregarding their voices and needs.

A further challenge for environmental justice, in terms of procedural justice, arises from a community's engagement in the adaptation process. We have shown that direct risk does not capture the full picture of impacts and can significantly underestimate the number of people at risk. This means that people who are indirectly affected may not be aware of the possible impacts and, therefore, will be less likely to engage and may even be resistant or hostile to adaptation actions, resulting in deteriorating community cohesion and trust. As events emerge and an eventual understanding of indirect risk is realized, residents with resources are likely to leave the area (Beck, 1992). This may result in areas at risk of isolation undergoing a transition over time toward a higher level of deprivation (Ratnadiwakara & Venugopal, 2020). To avoid this future transition state, the identification and monitoring of indirectly impacted communities must begin early. Early identification and engagement will ensure future actions and governmental support is considered and distributed more equitably across directly and indirectly impacted communities.

Further to understanding how risk is exacerbated when including indirect impacts is the need to understand how this change in risk profile is distributed among different sociodemographic groups; that is, are inequalities being exacerbated? Understanding risks, both direct and indirect, can support an equitable engagement and intervention process across different sociodemographic groups experiencing unequal risks. Figure 2 demonstrates how at a national level, existing inequalities in risk between ethnic groups are exacerbated. That is, both Māori and NZ/European populations are already disproportionately impacted when considering direct risk. When considering indirect risk, the impacted Māori population is estimated to increase from 13,010 to 30,387 during a present‐day one in 100 year event. Māori populations are overrepresented across both direct and indirect impacts when compared to other ethnic groups. Both Pacific and Asian population groups are seen to be consistently underrepresented in the exposed populations which may be attributed to their high occupancy rates in urban areas (Cook et al., 1999; Taylor, 2008). Interestingly, this respective state of over‐ and underrepresentation remains surprisingly stable at the national level over time, even as the absolute number of people impacted increases significantly. However, we note variations at local levels due to factors such as local topography, existing infrastructure, and community‐specific demographic shifts. One additional consideration is the intersectionality of demographics, as shown in Figure 1B, which identifies the most overrepresented demographic groups across income, area deprivation, and ethnicity and demonstrates the level of nuance that can be achieved through this analysis to find overlapping collectives of individuals that can have subsequent engagement and interventions tailored to their specific background and needs.

Figures 1 and 4 show how the distribution of inequality changes with the inclusion of indirect impacts is highly place‐specific, highlighting the importance of this analysis in order to find appropriate and equitable interventions. When we consider different sociodemographic characteristics such as income and socioeconomic deprivation, both partial indicators of adaptative capacity, Figures 3 and 4 show that the relative unequal distribution of burdens can be attenuated when including indirect impacts at the national level. However, in both cases, the underserved population is still overrepresented within the impacted population. At a localized level (Figure 4C), the inequality can be seen to be exacerbated by the consideration of indirect impacts. Given the nuanced and context‐specific nature of distributional justice through both direct and indirect impacts, it is critical that future risk assessments attempt to understand the distribution of both direct and indirect impacts if targeted intervention options are to be successful.

## 5 CONCLUSION

Communities around the world are beginning to assess their increasing risk driven by climate change and natural hazards and, as a result, are making consequential and long‐term investment decisions based on scenario‐based risk assessments. Our study enhances these assessments by examining distributional justice in the context of both direct and indirect impacts from natural hazards. We demonstrate that the inclusion of indirect impacts, such as isolation, can dramatically increase the number of people identified as being at risk, often by orders of magnitude. Furthermore, our analysis reveals that the distribution of these impacts varies significantly across different sociodemographic groups and spatial scales, with some inequalities exacerbated and others attenuated when indirect impacts are considered, highlighting the complex nature of distributional justice in climate risk. This finding underscores the need for adaptation planning to consider the full spectrum of consequences faced by communities, particularly in terms of how these impacts are distributed across different sociodemographic groups.

The nuanced and context‐specific nature of distributional justice in the face of climate‐related and natural hazards emphasizes the critical importance of enabling decision‐makers to assess the full range of risks and their respective distributions across different sociodemographic groups so that regardless of the equality of risk distribution, equitable responses and intervention decisions can still be made. While this paper focuses specifically on distributional justice, we suggest that both procedural and recognitional justice can be better considered by integrating measures of indirect risk (and its distribution) in future risk assessments.

Our approach leverages the recent advances in computational methodologies for measuring risk and accessibility as well as commonly available data (e.g., census) to demonstrate the practicability of measuring indirect risk and its associated disparity. This ultimately enables risk practitioners to evaluate and communicate the disparity within both direct and indirect risk. This is critical as it allows decision‐makers to identify the previously unidentified at‐risk communities, understand how risk is distributed across different groups, and ensure future actions are not maladaptive, ineffective, or inequitable.

Failure to account for the distribution of burdens can perpetuate existing disparities and further marginalize underserved communities. Our study has shown that trends in the distribution of risk are, in most cases, unpredictable due to the multiple variables involved. Hence, it is crucial to incorporate an analysis of distributional justice in all risk assessments to ensure that policies and interventions effectively address the varying disproportionate burdens faced by underserved groups.

## AUTHOR CONTRIBUTIONS

**M. J. Anderson**: Conceptualization; data curation; investigation; methodology; visualization; writing—original draft; writing—review editing. **L. Conrow**: Supervision; investigation; writing—review editing. **M. Hobbs**: Supervision; investigation; writing—review editing. **R. Paulik**: Data curation; writing—review editing. **P. Blackett**: Writing—review editing. **T. Logan**: Supervision; conceptualization; investigation; visualization; writing—review editing.

## CONFLICT OF INTEREST STATEMENT

MA & TL declare interests in a risk software and consulting company, Urban Intelligence.

## Figures and Tables

**FIGURE A.1:**
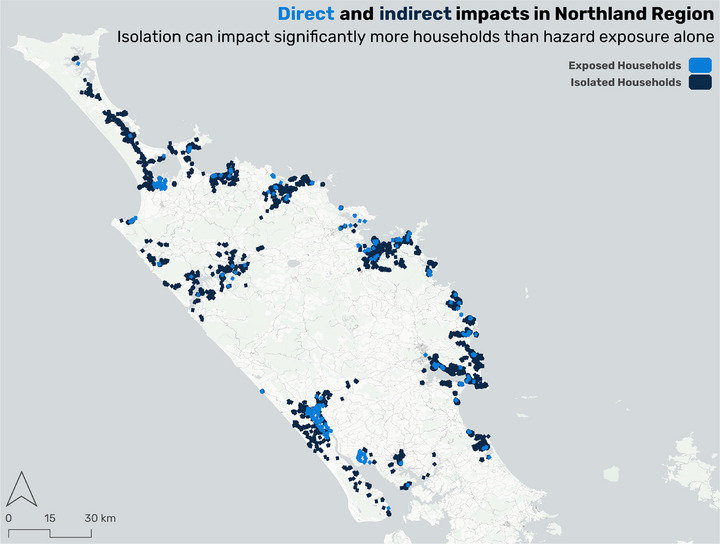
Spatial distribution of direct and indirect impacts under a present‐day 1% AEP coastal flood event.

## APPENDIX A

### A.1. Spatial distribution of direct and indirect impacts: Regional example

Figure A.1 demonstrates the spatial distribution of directly exposed and indirectly isolated households in the Northland Region of New Zealand under a present‐day 1% AEP (annual exceedance probability) coastal flood event. The map reveals a significant increase in affected households when considering indirect impacts, highlighting areas at risk of isolation that appear unaffected when only examining direct flood exposure. This localized example emphasizes the importance of evaluating both direct and indirect impacts in risk assessments to capture the full scope of a hazard's consequences and enable effective planning and adaptation efforts.

### A.3. Full result summary by territorial authority

**TABLE A.1:** Summary of results by territorial authority. Results are shown for a one in 100‐year coastal flooding event with 0 cm sea ‐level rise.

Area	Study population	Exposed population	Isolated population	Total impacted population	Total percent increase	Most overrepresented		
						Ethnicity	Deprivation index	Household income
**Coastal New Zealand**	**4,414,410**	**61,993**	**155,009**	**217,002**	**250**	**European**	**4–7**	**40–90k**
**Waitaki District**	22,268	1	91	92	9100	European	4–7	40–90k
**Wairoa District**	8849	6	412	418	6866.7	Māori	8–10	40–90k
**South Wairarapa District**	11,381	3	81	84	2700	European	4–7	40–90k
**Otorohanga District**	5724	16	367	383	2293.8	Māori	8–10	0–40k
**Whangarei District**	94,599	715	14,544	15,259	2034.1	European	1–3	40–90k
**Wellington City**	201,177	26	388	414	1492.3	European	1–3	90k+
**Far North District**	63,624	974	13,169	14,143	1352.1	European	8–10	0–40k
**Western Bay of Plenty District**	59,720	856	11,442	12,298	1336.7	Pacific	8–10	40–90k
**Opotiki District**	8595	48	574	622	1195.8	European	4–7	40–90k
**Whanganui District**	46,716	15	154	169	1026.7	Māori	4–7	0–40k
**New Plymouth District**	78,897	14	143	157	1021.4	European	1–3	90k+
**Tararua District**	13,145	26	238	264	915.4	Māori	8–10	40–90k
**Southland District**	24,872	96	813	909	846.9	Māori	8‐10	40‐90k
**Grey District**	12,701	195	1263	1458	647.7	European	4–7	40–90k
**Waitomo District**	10,941	92	568	660	617.4	European	4–7	0–40k
**Dunedin City**	125,798	796	4844	5640	608.5	European	1–3	90k+
**Lower Hutt City**	103,019	820	4891	5711	596.5	European	1–3	90k+
**Auckland**	1,535,193	4988	28,455	33,443	570.5	European	1–3	40–90k
**Thames‐Coromandel District**	31,203	4029	22,721	26,750	563.9	Māori	4–7	0–40k
**Porirua City**	55,559	79	353	432	446.8	European	1‐3	90k+
**Gisborne District**	45,981	117	467	584	399.1	Māori	8–10	40–90k
**Tauranga City**	127,580	2638	8003	10,641	303.4	Māori	4–7	40–90k
**Nelson City**	50,132	1721	5095	6816	296	European	1–3	90k+
**Hurunui District**	9966	51	144	195	282.4	European	4–7	40–90k
**Clutha District**	13,973	210	561	771	267.1	European	4–7	40–90k
**Kaipara District**	20,739	2245	5986	8231	266.6	European	4–7	0–40k
**Waikato District**	84,956	715	1750	2465	244.8	European	8–10	40–90k
**Matamata‐Piako District**	32,166	45	93	138	206.7	Asian	4–7	40–90k
**Masterton District**	25,404	36	74	110	205.6	European	4–7	40–90k
**Kaikoura District**	3717	147	286	433	194.6	Pacific	4–7	40–90k
**Westland District**	9,158	163	314	477	192.6	European	4–7	0–40k
**Tasman District**	53,054	1217	2313	3530	190.1	Māori	4–7	0–40k
**Invercargill City**	51,536	2787	5290	8077	189.8	Māori	1–3	90k+
**South Taranaki District**	26,022	13	18	31	138.5	European	8–10	40–90k
**Kapiti Coast District**	52,767	29	34	63	117.2	European	1–3	90k+
**Waipa District**	41,094	10	10	20	100	Māori	4–7	40–90k
**Selwyn District**	58,833	199	191	390	96	European	4–7	0–40k
**Christchurch City**	362,526	10,992	9795	20,787	89.1	European	4–7	40–90k
**Marlborough District**	46,533	140	124	264	88.6	Other	1–3	90k+
**Whakatane District**	35,745	2987	2407	5394	80.6	European	1–3	90k+
**Buller District**	7553	1453	854	2307	58.8	European	8–10	0–40k
**Central Hawke”s Bay District**	11,087	58	26	84	44.8	European	4–7	40–90k
**Hastings District**	79,520	1710	727	2437	42.5	European	4–7	40–90k
**Waimakariri District**	61,157	2136	804	2940	37.6	Māori	8–10	40–90k
**Hauraki District**	15,917	3491	945	4436	27.1	European	4–7	40–90k
**Napier City**	64,016	12,761	3177	15,938	24.9	Māori	8–10	40–90k
**Horowhenua District**	31,304	105	11	116	10.5	European	4–7	40–90k
**Manawatu District**	25,541	0	2	2	—	European	4–7	40–90k
**Gore District**	1835	0	0	0	0	—	—	—
**Upper Hutt City**	45,314	0	0	0	0	—	—	—
**Rangitikei District**	12,722	0	0	0	0	—	—	—
**Carterton District**	6878	0	0	0	0	—	—	—
**Waimate District**	7344	0	0	0	0	—	—	—
**South Waikato District**	7163	0	0	0	0	—	—	—
**Stratford District**	12,122	0	0	0	0	—	—	—
**Rotorua District**	44,534	0	0	0	0	—	—	—
**Ashburton District**	26,960	0	0	0	0	—	—	—
**Mackenzie District**	212	0	0	0	0	—	—	—
**Kawerau District**	5,960	0	0	0	0	—	—	—
**Ruapehu District**	1512	0	0	0	0	—	—	—
**Hamilton City**	149,396	0	0	0	0	—	—	—
**Timaru District**	46,389	0	0	0	0	—	—	—
**Palmerston North City**	84,017	0	0	0	0	—	—	—
